# Explaining Ionic Liquid Water Solubility in Terms of Cation and Anion Hydrophobicity

**DOI:** 10.3390/ijms10031271

**Published:** 2009-03-18

**Authors:** Johannes Ranke, Alaa Othman, Ping Fan, Anja Müller

**Affiliations:** 1 Abteilung Nachhaltigkeit in der Chemie, UFT Zentrum für Umweltforschung und Umwelttechnologie, Universität Bremen, Postfach 330440, 28334 Bremen, Germany; E-Mail: pingfan@web.de; 2 Medical biochemistry department, Kasr Alainy faculty of medicine Cairo University, Cairo, Egypt; E-Mail: alaaothman@hotmail.com; 3 School of Engineering and Science, Jacobs University Bremen, Postfach 750561, 28725 Bremen, Germany; E-Mail: anj.mueller@jacobs-university.de

**Keywords:** Ionic liquids, Water solubility, Cations, Anions, Lipophilicity

## Abstract

The water solubility of salts is ordinarily dictated by lattice energy and ion solvation. However, in the case of low melting salts also known as ionic liquids, lattice energy is immaterial and differences in hydrophobicity largely account for differences in their water solubility. In this contribution, the activity coefficients of ionic liquids in water are split into cation and anion contributions by regression against cation hydrophobicity parameters that are experimentally determined by reversed phase liquid chromatography. In this way, anion hydrophobicity parameters are derived, as well as an equation to estimate water solubilities for cation-anion combinations for which the water solubility has not been measured. Thus, a new pathway to the quantification of aqueous ion solvation is shown, making use of the relative weakness of interactions between ionic liquid ions as compared to their hydrophobicities.

## 1. Introduction

The presumed discovery of the first ionic liquid by Walden [1] took place in 1914. After that, it took over 80 years for the bloom of ionic liquid research to appear, starting with the discovery of a new class of air and water stable ionic liquids by Wilkes in 1992 [2]. It will maybe take another 80 years to realize the full potential of these substances, or, as Uwe Vagt (BASF) put it at the Intertech Pira Conference on Ionic Liquids in Prague in October 2007, “We are still at the beginning.” Numerous contributions [3, 4, 5, 6, 7, 8] have increased our understanding of basic properties, and to predict physical [9, 10, 11, 12] or toxicological [13, 14, 15] properties of ionic liquids. Especially in the context of green and sustainable chemistry, there is a vision of a molecular design of ionic liquids which would combine technical advantages with a set of favorable properties like ready availability and low risk for health and environment [16, 17].

The quality of our recently found correlation between cation hydrophobicity and cytotoxicity for ionic liquids with relatively small anions [14] inspired us to also investigate the correlation of cation hydrophobicity with water solubility data at 293 K that had previously been generated in our labs. Because of the good correlation found within this dataset (see below), we complemented our water solubility data with data from the scientific literature.

The model we use for the water solubility of ionic liquids is:

(1) $$\text{log}x_{\text{IL},\text{w}}^{\text{sat}}\;=\;-m\text{log}\;k_{0,\text{c}}\;+\;c_{a}\;+\;ɛ,$$

where $x_{\text{IL},\text{w}}^{\text{sat}}$ is the mole fraction solubility of the ionic liquid at 293 K, *k*_0,c_ is the cation hydrophobicity parameter as defined previously [14], *c*_a_ is an anion specific constant expressing their hydrophobicity, and *ε* is the random variable describing deviations from the model with unknown or unspecified causes. The slope *m* should ideally be unity if all model assumptions are satisfied.

Briefly, the model is based on the idea that the excess molar free energy of dissolving the ionic liquid in water Δ_IL,w_*g*^E^ = *RT* ln γ_IL,w_ can be expressed as the sum of a cation and an anion contribution, where the contribution of the cation is given by log *k*_0,c_ plus an unknown constant, and the contribution of the anion is extracted by a least squares fit of the model to the data.

## 2. Theory

For the theoretical discussion of the model we need a notation that covers both a) cation partitioning in reversed phase HPLC, quantified by the capacity factor *k*_c_ extrapolated to the conditions at the beginning of an acetonitrile gradient and b) the ionic liquid - water equilibrium at saturation, specified by the equilibrium mole fraction of ionic liquid in the water phase $x_{\text{w}}^{\text{sat}}$.

We first denote the chemical potential of an ionic liquid in solvent *s* as:

(2) $$\mu_{\text{IL},\text{s}}\;=\;\mu_{\text{IL}}^{•}\;+\;RT\text{ln}\;\gamma_{\text{IL},\text{s}}\;x_{\text{IL},\text{s}}$$

using the pure ionic liquid as the standard state. Restricting our model to ionic liquids that are composed of one cation type and one anion type at a 1:1 ratio (covering the majority of what is called ionic liquids today), we split *μ*_IL,s_ into the sum of the chemical potentials of cation and anion:

(3) $$\mu_{\text{IL},\text{s}}\;=\;\mu_{\text{c},\text{s}}\;+\;\mu_{\text{a},\text{s}}$$

with

(4) $$\mu_{\text{c},\text{s}}\;=\;\mu_{\text{c}}^{•}\;+\;RT\text{ln}\;\gamma_{\text{c},\text{s}}\;x_{\text{c},\text{s}}$$

(5) $$\mu_{\text{a},\text{s}}\;=\;\mu_{\text{a}}^{•}\;+\;RT\text{ln}\gamma_{\text{a},\text{s}}\;x_{\text{a},\text{s}}$$

using the pure ionic liquid as the standard state as in equation 2. Note that these ion chemical potentials are not defined *sensu strictu*, because ions of one polarity can not be solvated without the presence of suitable counteranions. However, for our discussion it is convenient to use the terminology of chemical potentials and activity coefficients of ions, although extrathermodynamic assumptions are necessary to determine them [18].

**Figure 1.**
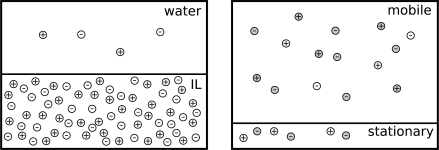
Equilibria of ionic liquid and water (left) and of ionic liquid cations between stationary and mobile phase in buffered reversed phase HPLC (right). Open circles are ionic liquid cations and anions, grey circles are buffer ions present in the HPLC mobile phase. In the case of the water saturation, electroneutrality in the water can only be kept if the concentrations of IL cations and anions are equal. In the case of buffered HPLC, IL cations and anions can partition largely independent of each other if sufficient concentrations of counterions are present in both phases.

We will now use this notation in order to discuss the two scenarios mentioned above, which are depicted in Figure 1:

In the case of the saturation of water with an ionic liquid, electroneutrality dictates that *x*_c,w_ = *x*_a,w_ = *x*_IL,w_ in the aqueous phase *w*. Neglecting the amount of water partitioning into the IL ( $x_{\text{IL},\text{IL}}^{\text{sat}}\;\approx \;1$, Assumption 1), and assuming that it does not significantly influence the chemical environment of the IL ( $\gamma_{\text{IL},\text{IL}}^{\text{sat}}\;\approx \;1$, Assumption 2), the chemical potential of the IL at water saturation is approximately equal to the standard chemical potential of the standard state, i.e. $\mu_{\text{IL},\text{IL}}^{\text{sat}}\;\approx \;\mu_{\text{IL}}^{•}$.

At the point of the saturation equilibrium, the chemical potentials of the IL have to be equal, i.e.

(6) $$\mu_{\text{IL},\text{IL}}^{\text{sat}}\;=\;\mu_{\text{IL},\text{w}}^{\text{sat}}$$

Using the standard state as an approximation for the left hand side of equation 6, and equation 2 for its right hand side,

(7) $$\mu_{\text{IL}}^{•}\;=\;\mu_{\text{IL}}^{•}\;+\;RT\text{ln}\gamma_{\text{IL},\text{s}}\;x_{\text{IL},\text{s}}$$

we can rearrange the result to read

(8) $$RT\text{ln}x_{\text{IL},\text{w}}^{\text{sat}}\;=\;-RT\text{ln}\gamma_{\text{IL},\text{w}}$$

We now assume that the excess molar free energy of dissolving the IL in water Δ_IL,w_*g*^E^ = *RT* ln γ_IL,w_ can be expressed as the sum of a cation and an anion contribution (Assumption 3), i.e.

(9) $$RT\text{ln}x_{\text{IL},\text{w}}^{\text{sat}}\;=\;-RT\text{ln}\gamma_{\text{c},\text{w}}\;-\;RT\text{ln}\gamma_{\text{a},\text{w}}$$

or, if we divide by *RT* and move to base ten for the logarithmic expressions

(10) $$\text{log}x_{\text{IL},\text{w}}^{\text{sat}}\;=\;-\text{log}\gamma_{\text{c},\text{w}}\;-\;\text{log}\gamma_{\text{a},\text{w}}$$

which gives us a relation between cation and ion hydrophobicities, expressed by their activity coefficients in water, and water solubility.

If we look at the cation partitioning between mobile and stationary phases in reversed phase HPLC, we can assume that electroneutrality in both phases is guaranteed by a sufficient concentration of counteranions present in them (Assumption 4). This only appears reasonable if the buffer anion is sufficiently hydrophobic to be able to enter the stationary interphase at the chemically modified silica surface, or if there is a sufficient surface concentration of negatively charged sites with loosely bound counterions, for example dissociated silanol groups.

If this condition is satisfied, and if we neglect ion pairing between ionic liquid cations and anions (Assumption 5), we can denote the cation partitioning coefficient between stationary and mobile phase

(11) $$K_{\text{c},\text{stm}}\;=\;\frac{x_{\text{c},\text{st}}}{x_{\text{c},\text{m}}}\;=\;\frac{\gamma_{\text{c},\text{m}}}{\gamma_{\text{c},\text{st}}},$$

neglecting any electrostatic contribution to the partitioning. In logarithmic form, this can be written as

(12) $$\text{log}\;K_{\text{c},\text{stm}}\;=\;\text{log}\;\gamma_{\text{c},\text{m}}\;+\;c_{1}$$

where the *c*_1_ represents the contribution of the activity coefficient of the cation in the stationary phase, but can potentially also represent an electrostatic contribution, which would be independent of the type of the cation. If we assume that the activity coefficient of the cation in the stationary phase is approximately invariant for the range of cations studied (Assumption 6), *c*_1_ can be taken as a constant.

In a previous study, we have shown how a measure for such a partition coefficient *K*_c,stm_ can be extracted from retention times in gradient HPLC. More specifically, we use the capacity factor log *k*_0,c_ for the situation at the beginning of the gradient, i.e. for the situation where the concentration of organic modifier is zero, and the mobile phase only consists of an ammonium acetate buffer, as a measure for the cation partitioning coefficient *K*_c,stm0_, also at the beginning of the HPLC gradient [14].

In partition chromatography, the constant of proportionality c_2_ in the equation for the capacity factor *k*

(13) $$k\;=\;c_{2}K_{\text{stm}}$$

is just the phase ratio of stationary and mobile phase in the separation column. Since the volumes of stationary and mobile phase in reversed phase HPLC are ill-defined, we treat *c*_2_ as a constant without explicit physical interpretation.

Combining equations 12 and 13, we find that the log of the capacity factor log *k*_0,c_ is just the cation activity coefficient in the mobile phase *m*_0_ at the beginning of the gradient plus a constant

(14) $$\text{log}k_{0,\text{c}}\;=\;\text{log}\gamma_{{\text{c},\text{m}}_{0}}\;+\;c_{3}.$$

The constant *c*_3_ incorporates chemical interactions of the cations with the stationary phase, possibly an electrostatic contribution, and the correlate to the phase ratio *c*_2_ from equation 13.

Under the final assumption that the activity coefficient of the cation in pure water is approximately equal to its activity coefficient in the mobile phase at the beginning of the gradient, in our case an ammonium acetate buffer, (Assumption 7), we can combine equations 10 and 14 to

(15) $$\text{log}x_{\text{IL},\text{w}}^{\text{sat}}\;=\;-\text{log}k_{0,\text{c}}\;-\;\text{log}\gamma_{\text{a},\text{w}}\;+\;c_{3}$$

Therefore the slope *m* in the model equation 1 for the water solubility of ionic liquids repeated below is ideally equal to unity The anion constant *c*_a_ equals *c*_3_ — log γ_a,w_.

(16) $$\text{log}x_{\text{IL},\text{w}}^{\text{sat}}\;=\;-m\;\text{log}k_{0,\text{c}}\;+\;c_{\text{a}}\;+ɛ$$

## 3. Materials and Methods

**Ionic liquid nomenclature**. In this study, the acronym scheme established at the UFT Center for Environmental Research and Sustainable Technology, University of Bremen is used for cations as illustrated in Table 1. Generally, numbers refer to alkyl chains (as in IM14 where the numbers refer to one methyl and one alkyl substituent on the nitrogens of the imidazolium cation) similar to their use in Wiswesser line notation. However, sometimes they are also pragmatically used to designate the site of substitution, as in Py4-3Me, where “-3Me” indicates 3-methyl substitution of the N-butylpyridinium cation.

**Ionic liquids** listed in Table 2 were used as received from Merck KGaA, Darmstadt, Germany. The first three entries in the table were used for the determination of their cation hydrophobicities as specified below. The remaining ionic liquids were used in the determination of their water solubility.

**Cation hydrophobicity** parameters log *k*_c,0_ were taken from [14] and [19]. The log *k*_c,0_ parameters for the cations 1-ethyl-3-methylimidazolium, 1-(3-hydroxypropyl)pyridinium, and 4-(ethoxymethyl)-4-methylmorpholinium newly presented here were derived from capacity factors calculated from isocratic retention times on the same reversed phase HPLC column type (Polaris Ether C18 from Varian, 150 mm, 3 mm inner diameter, 5 m particle size). Specifically, for each mobile phase composition, i.e. 2.5, 5, 10, 15 and 20 % gradient grade acetonitrile complemented with 0.25 % acetic acid (p.a., both Fluka, Buchs, Switzerland), a linear correlation was established between capacity factors under these conditions and previously established [14, 19] log *k*_c,0_ values. For the three cations without such previously established values, the mean of the predictions based on these linear correlations was used as log *k*_c,0_ in Table 3.

**Determination of water solubility** was carried out based on OECD guideline 105 for testing of chemicals. After establishment of approximate water solubility by a preliminary visual test, six aliquots of 100 mL water were equilibrated with the amount of ionic liquid resulting in five-fold oversaturation according to the preliminary result in a water bath at 30 degrees C, and repeatedly mixed by thoroughly shaking them manually. After 24, 48, and 72 hours two aliquots were transferred to a water bath at 20 degrees C. Each of those aliquots was gently centrifuged after a second 24 h equilibration period in the second water bath, and the ionic liquid concentration in the water phase was determined by reversed phase HPLC. The experimental uncertainty of the water solubility determinations depends on the soluble concentration and is better for ionic liquids exhibiting higher water solubility. Generally, deviations should be lower than 0.1 log units.

**HPLC determination of ionic liquid concentrations** in the saturated solutions was carried out using isocratic reversed phase HPLC on a Hewlett Packard 1100 Series system, equipped with online degasser, variable wavelength UV detector and a Bruker esquire ESI-MS ion trap detector. For the UV-absorbing cations, a Polaris Ether column from Varian also used for the hydrophobicity determination was used, with 20 mM KH_2_PO_4_ and 3.9 mM H_3_PO_4_ on channel A and gradient grade acetonitrile on channel B, using a suitable percentage of acetonitrile for each cation, and UV detection at 212 nm. For the cations without UV absorption, the same acetic acid/acetonitrile eluent system used in the hydrophobicity determination was used isocratically on the Polaris Ether RP 18 column, in order to have better compatibility with the ESI interface, again adapting the acetonitrile percentage to the analyte cation. For the quantification by ESI-MS, only a concentration range of two decades at maximum could be used, because of the nonlinear relation between concentration and peak area. In both cases, the pH of the eluent has to be around 3, in order to avoid tailing and overly long retention times. Conversely, for the most hydrophilic cations, a MonoChrom MS column from Varian was used for quantification, in order to obtain sufficient retention.

Ionic liquid concentrations at saturation derived from cation analysis via HPLC-ESI-MS were double checked by determining peak areas of the hydrophobic anions bis(trifluoromethlysulfonyl)imide and trifluorotris(pentafluoroethyl)phosphate in the negative mode of the ion trap detector on the same column, but with only 0.1 % to 0.25 % acetic acid as the aqueous part and 60 to 70 % MeOH. They diverged by less than ten percent, and the mean of cation and anion concentrations was taken as the final result.

## 4. Results

The data analysed using the model derived above are listed in Table 3. Most of the cation hydrophobicity data has already been published elsewhere by our group [14, 19]. The water solubility data generated by us has not been published previously.

**Figure 2.**
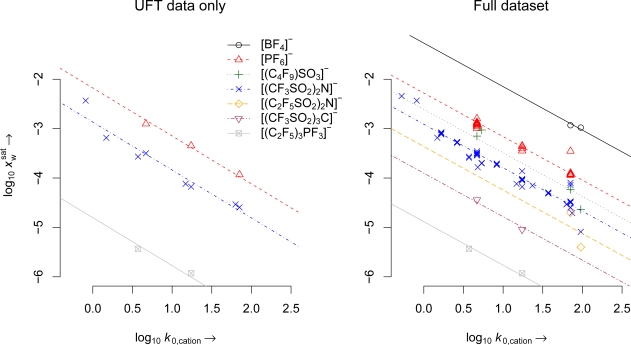
Plot of water solubility against log *k*_0,c_ of the cation of various ionic liquids that form a second phase with water at room temperature. To the left, only water solubility data generated in our labs are presented, to the right, water solubility data from the peer reviewed literature are included. Regression lines for constant anions have identical slopes −*m* as defined by model equation 1. Only data for anions with solubility data for at least two different cations are plotted.

In Figure 2, water solubilities of ionic liquids that form a second phase with water at room temperature are plotted against the hydrophobicity of their cation. The trend lines in the graph resulting from fitting the linear model specified in equation 1. Only data for anions combined with at least two different cations are shown.

The two available water solubility data for the (C_2_F_5_SO_2_)_2_N anion diverge by 0.7 log units, despite the similar hydrophobicity parameters of the two cations (Py8-4Me and IM18). Similar, but smaller deviations are found for the (C_2_F_5_SO_2_)_2_N salts of the same cations. It would be tempting to attribute these differences to H-bonding between the hydrogen in 2-position of the imidazolium ring and the sulfoxy groups of the bis(perfluoroalkylsulfonyl)imides in the pure ionic liquid, leading to smaller water solubilities of the imidazolium bis(perfluoroalkylsulfonyl)imides than predicted by their cation hydrophobicities. However, the size of the intralaboratory variability of the data as apparent in Table 5 in the Appendix does not really allow for such a conclusion, given that these data were generated by different groups.

In Table 4, the statistical parameters resulting from fitting this model to two sets of data are given. When modeling only the water solubility data from our labs (UFT only), the RSE was a little smaller than in the model for all solubility data (Full). This is not really surprising, as the equilibration procedure can have an impact on the results. The fact that the slope parameter *m* approximates the value expected according to the theoretical considerations is a striking evidence that both partitioning processes (ionic liquid/water equilibrium and cation partitioning in the HPLC column) are governed by the same cation property, even though the second phase is an ionic liquid in the one case, and a hydrophobic interphase at the surface of the chemically modified silica particles in the other case. The most plausible explanation is indeed to assume that this common property is their activity coefficient in aqueous systems with the pure ionic liquid as the reference state.

For the UFT only model, the slope *m* is 0.97, which is within the expected uncertainty for such a model. The larger deviation of *m* from unity in the full model can partly be explained by a large scatter of experimental values for different 1-octyl-3-methylimidazolium ionic liquids. The lower values better fit the theory, which might be caused by a disturbance of the chosen analytical method by the formation of microdroplets, which is also a known problem in the determination of 1-octanol/water partition coefficients.

The anion constants *c*_a_ in Table 4 can be seen as hydrophobicity measures, complementary to the log *k*_0,c_ values for the cations. The fact that many of these constants are derived from data for only one ionic liquid explains the very high coefficient of determination *R*^2^ for both models. It is worthy to note that the anion constants in Table 4 for the [PF_6_]^−^, [(CF_3_SO_2_)_2_N]^−^, and [(CF_3_SO_2_)_3_C]^−^ anions are very similar, regardless if only the UFT data are employed in the model building, or if they are supplemented by the more abundant and completely independent literature data.

A more direct determination of the anion hydrophobicity using an HPLC based method similar to the method of cation hydrophobicity determination used here was attempted. So far, the results have been found to be unreliable, presumably because of the limited suitability of the chromatographic partitioning system used.

The correlations presented here confound the validity of the log *k*_0,c_ values as derived from HPLC as a measure of the tendency of a cation to evade aqueous phases. Previously, we have shown that the cytotoxicity of ionic liquids can be described by the cation log *k*_0,c_ with an RSE value of 0.47 [14]. The much larger RSE in that former study can be attributed to a) the fact that several anions were included in one correlation, b) to the inclusion of the large aromatic quinolinium cations in the earlier study, and c) to the fact that biological test systems generally yield higher variabilities than physicochemical systems.

The applicability domain of the model for predicting water solubilities from this model is naturally restricted by the availability of log *k*_0,c_ values (see [16] and this study) and anion constants (Table 4). The accuracy of log *k*_0,c_ values is best for 0 < log *k*_0,c_ < 4, because of limitations of the determination method. In this interval, the uncertainty is estimated to be less than 0.1 log units. The estimated standard error of predictions of water solubility in this interval is 0.2 log units for the seven anions shown on the right side of Figure 2 (model for selected data). For the anions with only one data point, we advise to only extrapolate to ionic liquids with cations that have a log *k*_0,c_ that does not deviate more than 1 log unit from the log *k*_0,c_ of the given data point.

For the convenience of the reader, the water solubilities estimated by the full solubility model are listed together with the experimental data in Table 5 in the Appendix. Besides the explanations given above, no systematic deviations from the model were noted. In general, the database for the bis(trifluoromethylsulfonyl) imide ionic liquids is quite extensive, and there is a very good agreement between the majority of literature sources where more than one value was found for the same ionic liquid. However, there are several outlying data points e.g. for IM18 bis(trifluoromethylsulfonyl)imide, that can only be explained by a considerable interlaboratory variability.

The approach taken here is in some ways similar to the one taken by Kakiuchi and co-workers, explaining the water solubility of ionic liquids in terms of their ion transfer potentials between ionic liquid and water [23], for which the ion transfer potentials between nitrobenzene and water are proposed as an approximation [33]. This group provided experimental evidence for the assumption that the ion transfer potentials between ionic liquids and water for the hydrophobic ions is independent of the concentration of hydrophilic ions like Na, K and Cl in the aqueous phase. This can be taken as a corroboration of our assumption, that the activity coefficients of the hydrophobic cations are similar in water as in the mobile phase of our liquid chromatographic system (Assumption 7 in the supporting information). However, the reliability of prediction methods for the water solubility of ionic liquids based on such theories is limited by the quantity and precision of experimental data.

## 5. Conclusions

A new pathway to the approximate quantification of aqueous ion solvation is shown, making use of the relative weakness of interactions between ionic liquid ions as compared to their hydrophobicities. We believe that the excess free energies of IL solution in aqueous phases that can be estimated from log *k*_0,c_ and *c*_A_ values presented here are a valuable tool to quantitatively predict equilibrium concentrations of ionic liquids in aqueous phases, contributing to the toolbox necessary for a molecular design of ionic liquid applications as well as to a more thorough understanding of their partitioning behavior in general.

## Figures and Tables

**Table 1.** Chemical structures of the cations treated in this study, along with their acronyms. In the acronyms, IM, Mor, N, Pip, Pyr and Py are used for the imidazolium, morpholinium, ammonium, piperidinium, pyrrolidinium and pyridinium head groups. *n* designates linear alkyl chains with varying length, where *n* is the number of carbon atoms in the chain. In the graphs, these chains correspond to the residues R.

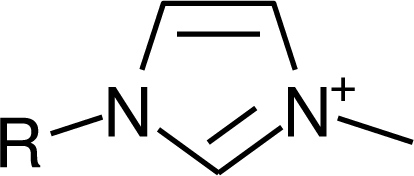 IM1*n*	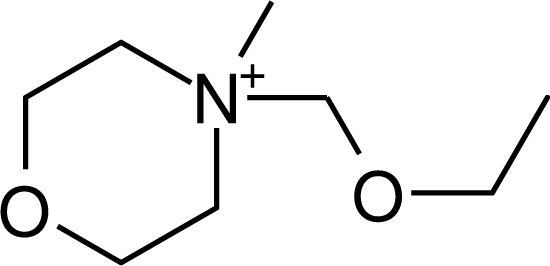 Mor11O2	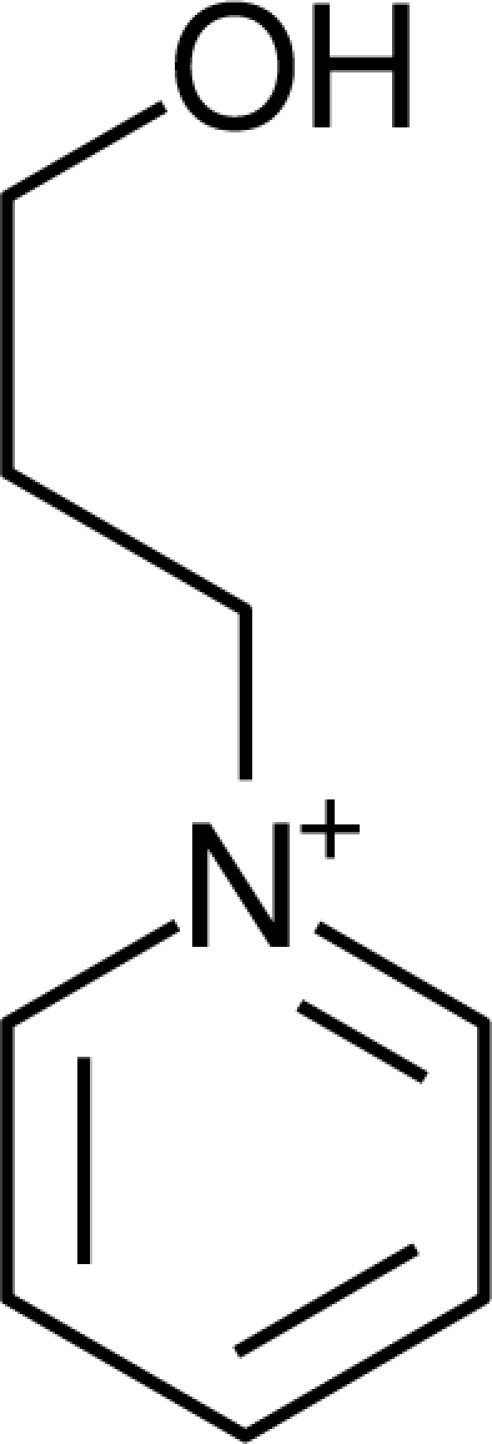 Py3OH
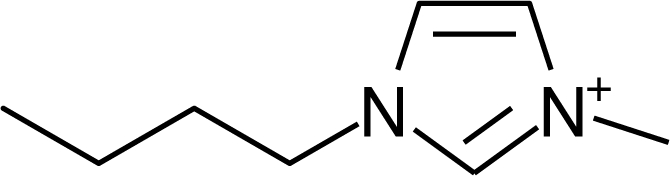 IM14	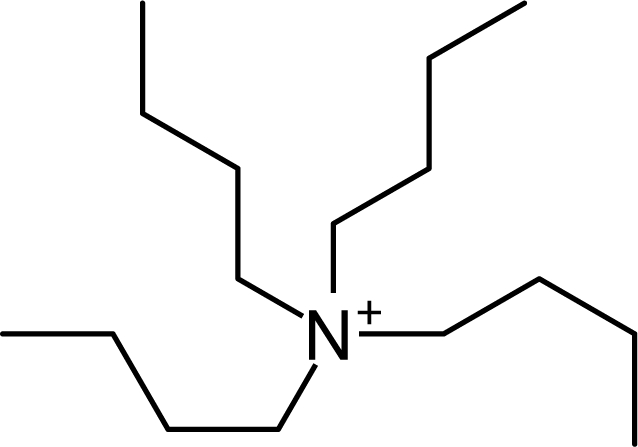 N4444	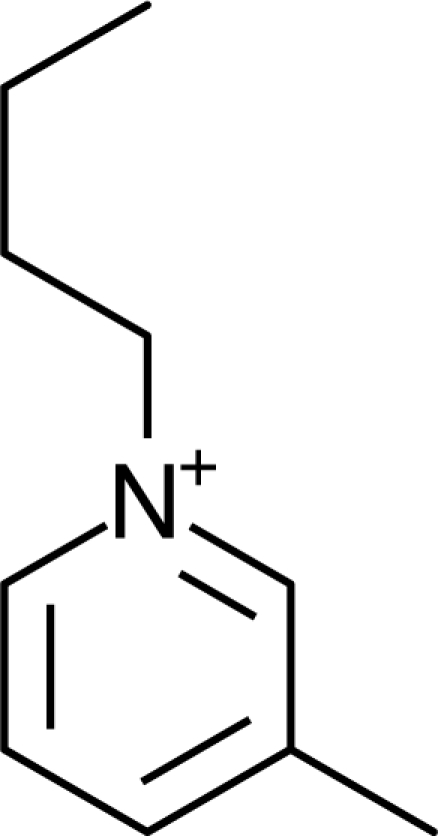 Py4-3Me
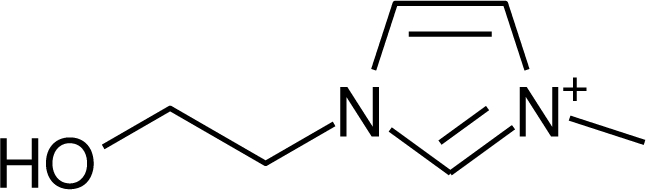 IM12OH	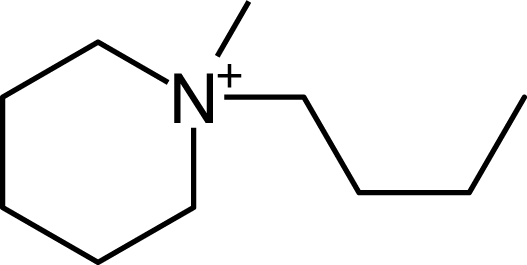 Pip14	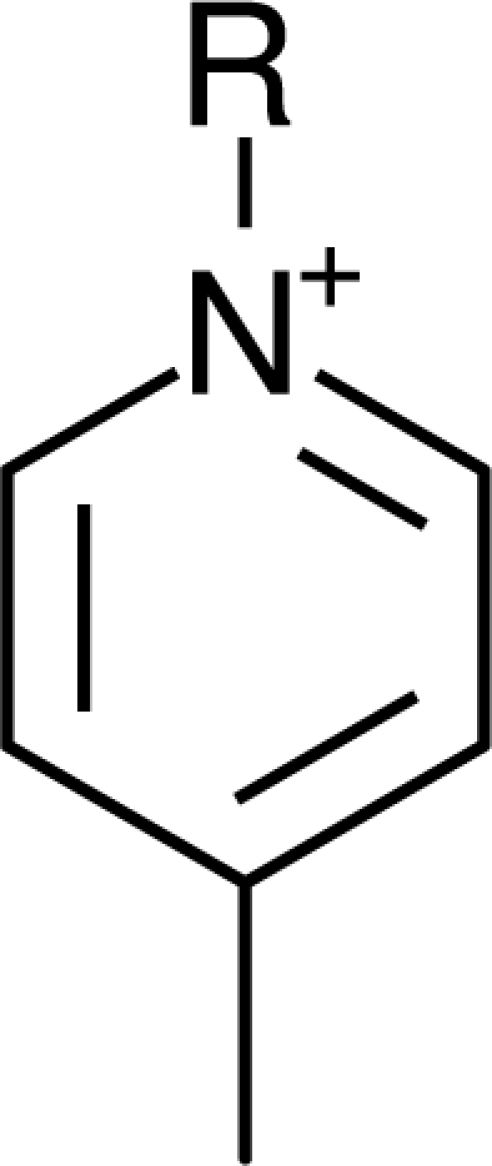 Py*n*-4Me
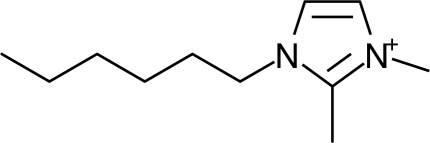 IM16-2Me	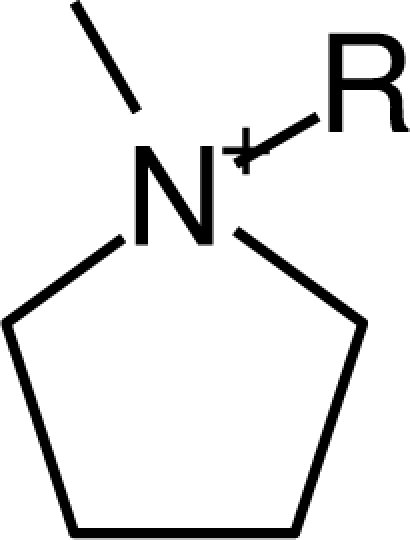 Pyr1*n*	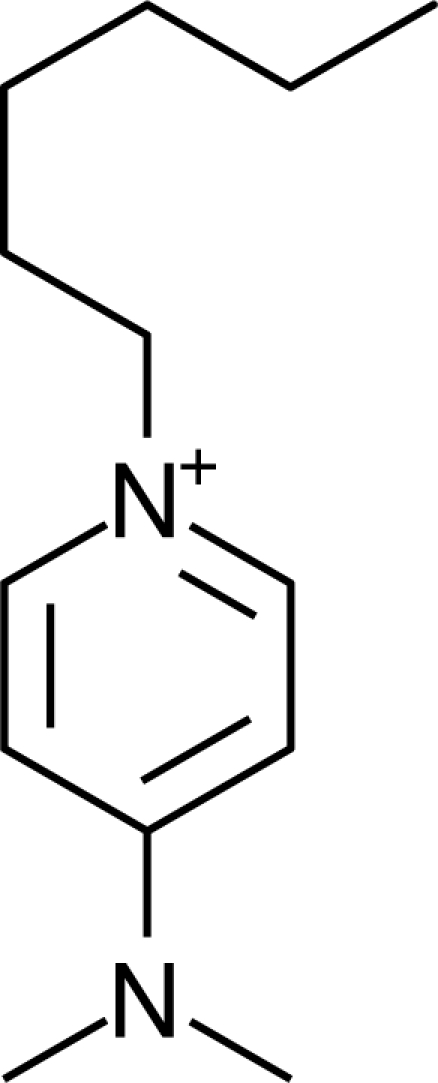 Py6-4NMe2

**Table 2.** Ionic liquids used for generation of original data for this study.

Ionic liquid	Acronym
1-ethyl-3-methylimidazolium chloride	IM12 Cl
1-(3-hydroxypropyl)pyridinium chloride	Py3OH Cl
4-(ethoxymethyl)-4-methylmorpholinium chloride	Mor11O2 Cl

1-butyl-3-methylimidazolium hexafluorophosphate	IM14 PF6
1-butyl-3-methylimidazolium bis(trifluoromethylsulfonyl)imide	IM14 (CF3SO2)2N
1-hexyl-3-methylimidazolium hexafluorophosphate	IM16 PF6
1-hexyl-3-methylimidazolium bis(trifluoromethylsulfonyl)imide	IM16 (CF3SO2)2N
1-hexyl-3-methylimidazolium tris(trifluoromethylsulfonyl)methide	IM16 (CF3SO2)3C
1-hexyl-3-methylimidazolium trifluorotris(pentafluoroethyl)phosphate	IM16 (C2F5)3PF3
1-octyl-3-methylimidazolium hexafluorophosphate	IM18 PF6
1-octyl-3-methylimidazolium bis(trifluoromethylsulfonyl)imide	IM18 (CF3SO2)2N
1-(3-hydroxypropyl)pyridinium bis(trifluoromethylsulfonyl)imide	Py3OH (CF3SO2)2N
4-(dimethylamino)-1-hexylpyridinium bis(trifluoromethylsulfonyl)imide	Py6-4NMe2 (CF3SO2)2N
1-butyl-1-methylpyrrolidinium bis(trifluoromethylsulfonyl)imide	Pyr14 (CF3SO2)2N
1-butyl-1-methylpyrrolidinium trifluorotris(pentafluoroethyl)phosphate	Pyr16 (C2F5)3PF3
1-hexyl-1-methylpyrrolidinium bis(trifluoromethylsulfonyl)imide	Pyr16 (CF3SO2)2N

**Table 3.** Water solubility at temperatures within 293.15 ± 5 K and cation hydrophobicity. Data without source attribution are published here for the first time. Data with a mole fraction solubility greater than 0.05 are not considered, because this would conflict with Assumptions 1 and 2 stated in the text. Data from Branco et al. [20] were not considered as they strongly diverge from other sources for an unknown reason.

	Cation hydrophobicity		IL water solubility	
Cation[a]	log *k*_0,c_	Anion[b]	${\text{log}}_{10}x_{\text{IL},\text{w}}^{\text{sat}}$	*T* [K]
IM16	1.2 [14]	(C_2_F_5_)_3_PF_3_	−5.93	293.15
Pyr14	0.57 [14]	(C_2_F_5_)_3_PF_3_	−5.43	293.15
Py8-4Me	2 [14]	(C_2_F_5_SO_2_)_2_N	−5.4	298.15 [21]
Py8-4Me	2 [14]	(CF_3_SO_2_)_2_N	−5.09	298.15 [21]
IM16	1.2 [14]	(CF_3_SO_2_)_3_C	−5.04	293.15
Py8-4Me	2 [14]	AsF_6_	−4.91	298.15 [21]
Pyr18	1.9 [14]	(CF_3_SO_2_)_2_N	−4.71	298 [22]
IM18	1.9 [14]	(C_2_F_5_SO_2_)_2_N	−4.7	298 [23]
Py8-4Me	2 [14]	(C_4_F_9_)SO_3_	−4.63	298.15 [21]
IM18	1.9 [14]	(CF_3_SO_2_)_2_N	−4.6	298 [23]
IM18	1.9 [14]	(CF_3_SO_2_)_2_N	−4.59	293.15
Py6-4NMe2	1.8 [19]	(CF_3_SO_2_)_2_N	−4.53	293.15
Py6-4NMe2	1.8 [19]	(CF_3_SO_2_)_2_N	−4.53	296.5 [24]
IM18	1.9 [14]	(CF_3_SO_2_)_2_N	−4.5	288.15 [25]
IM18	1.9 [14]	(CF_3_SO_2_)_2_N	−4.49	293.15 [25]
IM18	1.9 [14]	(CF_3_SO_2_)_2_N	−4.47	298.15 [25]
IM14	0.67 [14]	(CF_3_SO_2_)_3_C	−4.44	296.5 [24]
IM17	1.6 [14]	(CF_3_SO_2_)_2_N	−4.31	288.15 [25]
IM17	1.6 [14]	(CF_3_SO_2_)_2_N	−4.3	293.15 [25]
IM17	1.6 [14]	(CF_3_SO_2_)_2_N	−4.29	298.15 [25]
IM18	1.9 [14]	(C_4_F_9_)SO_3_	−4.23	298 [26]
IM16	1.2[14]	(CF_3_SO_2_)_2_N	−4.18	293.15
IM16-2Me	1.4 [14]	(CF_3_SO_2_)_2_N	−4.15	296.5 [24]
IM18	1.9 [14]	(CF_3_SO_2_)_2_N	−4.14	296.5 [24]
Pyr16	1.2 [14]	(CF_3_SO_2_)_2_N	−4.12	293.15
IM18	1.9 [14]	(CF_3_SO_2_)_2_N	−4.1	298 [26]
IM18	1.9 [14]	(CF_3_SO_2_)_2_N	−4.1	293.15 [27]
IM16	1.2 [14]	(CF_3_SO_2_)_2_N	−4.05	288.15 [25]
IM16	1.2 [14]	(CF_3_SO_2_)_2_N	−4.05	293.15 [25]
IM16	1.2 [14]	(CF_3_SO_2_)_2_N	−4.03	296.5 [24]
IM16	1.2 [14]	(CF_3_SO_2_)_2_N	−4.02	298.15 [25]
IM18	1.9 [14]	PF_6_	−3.95	288.15 [28]
IM18	1.9 [14]	PF_6_	−3.93	293.15
IM18	1.9 [14]	PF_6_	−3.92	293.15 [28]
IM18	1.9 [14]	PF_6_	−3.9	298.15 [28]
IM16	1.2 [14]	(CF_3_SO_2_)_2_N	−3.86	293.15 [27]
Pip14	0.68 [19]	(CF_3_SO_2_)_2_N	−3.78	298 [22]
IM15	0.92 [14]	(CF_3_SO_2_)_2_N	−3.74	288.15 [25]
IM15	0.92 [14]	(CF_3_SO_2_)_2_N	−3.73	293.15 [25]
IM15	0.92 [14]	(CF_3_SO_2_)_2_N	−3.71	298.15 [25]
Py4-3Me	0.73 [14]	(CF_3_SO_2_)_2_N	−3.7	296.5 [24]
Py4-4Me	0.73 [14]	(CF_3_SO_2_)_2_N	−3.69	298 [26]
Py8-4Me	2 [14]	PhBF_3_	−3.6	298.15 [21]
Pyr14	0.57 [14]	(CF_3_SO_2_)_2_N	−3.59	298 [22]
Pyr14	0.57 [14]	(CF_3_SO_2_)_2_N	−3.57	293.15
IM14	0.67 [14]	(CF_3_SO_2_)_2_N	−3.54	288.15 [25]
IM14	0.67 [14]	(CF_3_SO_2_)_2_N	−3.53	293.15 [25]
IM14	0.67 [14]	(CF_3_SO_2_)_2_N	−3.51	298.15 [25]
IM14	0.67 [14]	(CF_3_SO_2_)_2_N	−3.51	298 [26]
IM14	0.67 [14]	(CF_3_SO_2_)_2_N	−3.5	296.5 [24]
IM14	0.67 [14]	(CF_3_SO_2_)_2_N	−3.5	293.15
IM14	0.67 [14]	(CF_3_SO_2_)_2_N	−3.49	294.15 [29]
IM14	0.67 [14]	(CF_3_SO_2_)_2_N	−3.46	293.15 [27]
IM18	1.9 [14]	PF_6_	−3.46	295 [30]
IM16	1.2 [14]	PF_6_	−3.45	288.15 [28]
IM16	1.2 [14]	PF_6_	−3.41	293.15 [28]
IM16	1.2 [14]	PF_6_	−3.36	298.15 [28]
IM16	1.2 [14]	PF_6_	−3.35	293.15
IM13	0.42 [14]	(CF_3_SO_2_)_2_N	−3.29	288.15 [25]
IM13	0.42 [14]	(CF_3_SO_2_)_2_N	−3.28	293.15 [25]
IM13	0.42 [14]	(CF_3_SO_2_)_2_N	−3.27	298.15 [25]
Mor11O2	0.17	(CF_3_SO_2_)_2_N	−3.19	293.15
IM14	0.67 [14]	(C_4_F_9_)SO_3_	−3.15	298 [26]
IM12	0.22	(CF_3_SO_2_)_2_N	−3.12	288.15 [25]
IM12	0.22	(CF_3_SO_2_)_2_N	−3.1	296.5 [24]
IM12	0.22	(CF_3_SO_2_)_2_N	−3.1	293.15 [25]
Py8-4Me	2 [14]	CF_3_SO_3_	−3.09	298.15 [21]
IM12	0.22	(CF_3_SO_2_)_2_N	−3.08	293.15 [27]
IM12	0.22	(CF_3_SO_2_)_2_N	−3.08	298.15 [25]
Py4-4Me	0.73 [14]	(C_4_F_9_)SO_3_	−3.03	298 [26]
IM14	0.67 [14]	PF_6_	−3	288.15 [28]
Py8-4Me	2 [14]	BF_4_	−2.98	298.15 [21]
IM14	0.67 [14]	PF_6_	−2.96	293.15 [28]
IM14	0.67 [14]	PF_6_	−2.93	294 [31]
IM18	1.9 [14]	BF_4_	−2.93	295 [30]
IM14	0.67 [14]	PF_6_	−2.92	298.15 [28]
IM14	0.67 [14]	PF_6_	−2.9	293.15
IM14	0.67 [14]	PF_6_	−2.9	296.5 [24]
IM14	0.67 [14]	PF_6_	−2.89	295 [30]
IM14	0.67 [14]	PF_6_	−2.87	294.15 [29]
IM14	0.67 [14]	PF_6_	−2.8	293.15 [27]
IM12	0.22	B(CN)_4_	−2.46	296.5 [24]
Py3OH	−0.09	(CF_3_SO_2_)_2_N	−2.43	293.15
IM12OH	−0.28 [19]	(CF_3_SO_2_)_2_N	−2.34	296.5 [24]
N4444	2.3 [14]	(6-2Et)2SS	−1.52	298 [32]

[a] Cation acronyms explained in Table 1

[b] (6-2Et)-2SS = bis(2-ethylhexyl)sulfosuccinate

**Table 4.** Statistical parameters of the model described in equation 1 applied to only our own water solubility data at 293.15 K (UFT data only), and the complete water solubility dataset for 293.15 ± 5 K (Full dataset) given in Table 3. Numbers in parentheses after the anion constants are the number of data points for each anion that the constant is based on. *n* is the overall number of data points in the model, df is the number of statistical degrees of freedom, *R*^2^ is the fraction of the variability in water solubility explained by the model, and RSE is the standard error of the residuals. Note that data points for anions that are only present in one IL have a zero residual, giving a favorable bias to *R*^2^ and RSE. The anion acronym (6-2Et)2SS stands for bis(2-ethylhexyl)sulfosuccinate.

Parameter	UFT data only	Full dataset
*m*	0.97	0.881
*^c^*(6-2Et)2SS		0.521 (1)
*^c^*BF4		−1.268 (2)
*^c^*CF3SO3		−1.343 (1)
*^c^*C(CN)3		−1.642 (3)
*^c^*PhBF3		−1.853 (1)
*^c^*B(CN)4		−2.264 (1)
*^c^*PF6	−2.178 (3)	−2.28 (18)
*^c^*(C4F9)SO3		−2.61 (4)
*^c^*(CF3SO2)2N	−2.868 (8)	−2.911 (50)
*^c^*AsF6		−3.165 (1)
*^c^*(C2F5SO2)2N		−3.363 (2)
*^c^*(CF3SO2)3C	−3.841 (1)	−3.902 (2)
*^c^*(C2F5)3PF3	−4.803 (2)	−4.883 (2)

*n*	14	88
df	9	74
*R*^2^	0.999	0.998
RSE	0.157	0.16

## Appendix

**Table 5.** Overview of experimental (superscript exp) and estimated (superscript est) water solubility data, sorted by the estimated values. Δ_est,exp_ is the difference between estimated and observed values on a log scale. The model based on all suitable data was used for the estimation.

Cation[a]	Anion[b]	${\text{log}}_{10}x_{\text{IL},\text{w}}^{\text{sat},\text{exp}}$	${\text{log}}_{10}x_{\text{IL},\text{w}}^{\text{sat},\text{est}}$	Δ_est,exp_	Temperature [K]
IM16	(C_2_F_5_)_3_PF_3_	−5.93	−5.98	−0.05	293.15
Pyr14	(C_2_F_5_)_3_PF_3_	−5.43	−5.39	0.04	293.15
Py8-4Me	(C_2_F_5_SO_2_)_2_N	−5.4 [21]	−5.11	0.29	298.15
IM16	(CF_3_SO_2_)_3_C	−5.04	−4.99	0.05	293.15
IM18	(C_2_F_5_SO_2_)_2_N	−4.7 [23]	−4.99	−0.29	298
Py8-4Me	AsF_6_	−4.91 [21]	−4.91	0	298.15
Py8-4Me	(CF_3_SO_2_)_2_N	−5.09 [21]	−4.66	0.43	298.15
Pyr18	(CF_3_SO_2_)_2_N	−4.71 [22]	−4.56	0.15	298
IM18	(CF_3_SO_2_)_2_N	−4.5 [25]	−4.54	−0.04	288.15
IM18	(CF_3_SO_2_)_2_N	−4.59	−4.54	0.05	293.15
IM18	(CF_3_SO_2_)_2_N	−4.1 [27]	−4.54	−0.44	293.15
IM18	(CF_3_SO_2_)_2_N	−4.49 [25]	−4.54	−0.05	293.15
IM18	(CF_3_SO_2_)_2_N	−4.14 [24]	−4.54	−0.4	296.5
IM18	(CF_3_SO_2_)_2_N	−4.1 [26]	−4.54	−0.44	298
IM18	(CF_3_SO_2_)_2_N	−4.6 [23]	−4.54	0.06	298
IM18	(CF_3_SO_2_)_2_N	−4.47 [25]	−4.54	−0.07	298.15
Py6-4NMe2	(CF_3_SO_2_)_2_N	−4.53	−4.5	0.03	293.15
Py6-4NMe2	(CF_3_SO_2_)_2_N	−4.53 [24]	−4.5	0.03	296.5
IM14	(CF_3_SO_2_)_3_C	−4.44 [24]	−4.49	−0.05	296.5
Py8-4Me	(C_4_F_9_)SO_3_	−4.63 [21]	−4.35	0.28	298.15
IM17	(CF_3_SO_2_)_2_N	−4.31 [25]	−4.29	0.02	288.15
IM17	(CF_3_SO_2_)_2_N	−4.3 [25]	−4.29	0.01	293.15
IM17	(CF_3_SO_2_)_2_N	−4.29 [25]	−4.29	0	298.15
IM18	(C_4_F_9_)SO_3_	−4.23 [26]	−4.24	−0.01	298
IM16-2Me	(CF_3_SO_2_)_2_N	−4.15 [24]	−4.12	0.03	296.5
IM16	(CF_3_SO_2_)_2_N	−4.05 [25]	−4	0.05	288.15
IM16	(CF_3_SO_2_)_2_N	−4.18	−4	0.18	293.15
IM16	(CF_3_SO_2_)_2_N	−3.86 [27]	−4	−0.14	293.15
IM16	(CF_3_SO_2_)_2_N	−4.05 [25]	−4	0.05	293.15
IM16	(CF_3_SO_2_)_2_N	−4.03 [24]	−4	0.03	296.5
IM16	(CF_3_SO_2_)_2_N	−4.02 [25]	−4	0.02	298.15
Pyr16	(CF_3_SO_2_)_2_N	−4.12	−3.94	0.18	293.15
IM18	PF_6_	−3.95 [28]	−3.91	0.04	288.15
IM18	PF_6_	−3.92 [28]	−3.91	0.01	293.15
IM18	PF_6_	−3.93	−3.91	0.02	293.15
IM18	PF_6_	−3.46 [30]	−3.91	−0.45	295
IM18	PF_6_	−3.9 [28]	−3.91	−0.01	298.15
IM15	(CF_3_SO_2_)_2_N	−3.74 [25]	−3.72	0.02	288.15
IM15	(CF_3_SO_2_)_2_N	−3.73 [25]	−3.72	0.01	293.15
IM15	(CF_3_SO_2_)_2_N	−3.71 [25]	−3.72	−0.01	298.15
Py8-4Me	PhBF_3_	−3.6 [21]	−3.6	0	298.15
Py4-3Me	(CF_3_SO_2_)_2_N	−3.7 [24]	−3.55	0.15	296.5
Py4-4Me	(CF_3_SO_2_)_2_N	−3.69 [26]	−3.55	0.14	298
Pip14	(CF_3_SO_2_)_2_N	−3.78 [22]	−3.51	0.27	298
IM14	(CF_3_SO_2_)_2_N	−3.54 [25]	−3.5	0.04	288.15
IM14	(CF_3_SO_2_)_2_N	−3.5	−3.5	0	293.15
IM14	(CF_3_SO_2_)_2_N	−3.53 [25]	−3.5	0.03	293.15
IM14	(CF_3_SO_2_)_2_N	−3.46 [27]	−3.5	−0.04	293.15
IM14	(CF_3_SO_2_)_2_N	−3.49 [29]	−3.5	−0.01	294.15
IM14	(CF_3_SO_2_)_2_N	−3.5 [24]	−3.5	0	296.5
IM14	(CF_3_SO_2_)_2_N	−3.51 [26]	−3.5	0.01	298
IM14	(CF_3_SO_2_)_2_N	−3.51 [25]	−3.5	0.01	298.15
Pyr14	(CF_3_SO_2_)_2_N	−3.57	−3.41	0.16	293.15
Pyr14	(CF_3_SO_2_)_2_N	−3.59 [22]	−3.41	0.18	298
IM16	PF_6_	−3.45 [28]	−3.37	0.08	288.15
IM16	PF_6_	−3.35	−3.37	−0.02	293.15
IM16	PF_6_	−3.41 [28]	−3.37	0.04	293.15
IM16	PF_6_	−3.36 [28]	−3.37	−0.01	298.15
IM13	(CF_3_SO_2_)_2_N	−3.29 [25]	−3.28	0.01	288.15
IM13	(CF_3_SO_2_)_2_N	−3.28 [25]	−3.28	0	293.15
IM13	(CF_3_SO_2_)_2_N	−3.27 [25]	−3.28	−0.01	298.15
Py4-4Me	(C_4_F_9_)SO_3_	−3.03 [26]	−3.25	−0.22	298
IM14	(C_4_F_9_)SO_3_	−3.15 [26]	−3.2	−0.05	298
IM12	(CF_3_SO_2_)_2_N	−3.12 [25]	−3.1	0.02	288.15
IM12	(CF_3_SO_2_)_2_N	−3.08 [27]	−3.1	−0.02	293.15
IM12	(CF_3_SO_2_)_2_N	−3.1 [25]	−3.1	0	293.15
IM12	(CF_3_SO_2_)_2_N	−3.1 [24]	−3.1	0	296.5
IM12	(CF_3_SO_2_)_2_N	−3.08 [25]	−3.1	−0.02	298.15
Py8-4Me	CF_3_SO_3_	−3.09 [21]	−3.09	0	298.15
Mor11O2	(CF_3_SO_2_)_2_N	−3.19	−3.06	0.13	293.15
Py8-4Me	BF_4_	−2.98 [21]	−3.01	−0.03	298.15
IM18	BF_4_	−2.93 [30]	−2.9	0.03	295
IM14	PF_6_	−3 [28]	−2.87	0.13	288.15
IM14	PF_6_	−2.8 [27]	−2.87	−0.07	293.15
IM14	PF_6_	−2.9	−2.87	0.03	293.15
IM14	PF_6_	−2.96 [28]	−2.87	0.09	293.15
IM14	PF_6_	−2.93 [31]	−2.87	0.06	294
IM14	PF_6_	−2.87 [29]	−2.87	0	294.15
IM14	PF_6_	−2.89 [30]	−2.87	0.02	295
IM14	PF_6_	−2.9 [24]	−2.87	0.03	296.5
IM14	PF_6_	−2.92 [28]	−2.87	0.05	298.15
Py3OH	(CF_3_SO_2_)_2_N	−2.43	−2.83	−0.4	293.15
IM12OH	(CF_3_SO_2_)_2_N	−2.34 [24]	−2.66	−0.32	296.5
IM12	B(CN)_4_	−2.46 [24]	−2.46	0	296.5
N4444	(6-2Et)2SS	−1.52 [32]	−1.52	0	298

[a] Cation acronyms explained in Table 1

[b] (6-2Et)-2SS = bis(2-ethylhexyl)sulfosuccinate
